# Ttk69-dependent repression of *lozenge *prevents the ectopic development of R7 cells in the *Drosophila *larval eye disc

**DOI:** 10.1186/1471-213X-9-64

**Published:** 2009-12-09

**Authors:** Nicole A Siddall, Gary R Hime, John A Pollock, Philip Batterham

**Affiliations:** 1 Department of Genetics, University of Melbourne, Parkville, Vic 3010, Australia; 2 Department of Anatomy and Cell Biology, University of Melbourne, Parkville, Vic 3010, Australia; 3 ARC Centre of Excellence in Biotechnology and Development, University of Newcastle, Callaghan, NSW 2308, Australia; 4 Department of Biological Sciences, Duquesne University, Pittsburgh, PA 15282, USA; 5 Centre for Environmental Stress and Adaptation Research, The University of Melbourne, Parkville, Vic 3010, Australia

## Abstract

**Background:**

During the development of the *Drosophila *eye, specific cell types differentiate from an initially equipotent group of uncommitted precursor cells. The *lozenge *(*lz*) gene, which is a member of the Runt family of transcriptional regulators, plays a pivotal role in mediating this process through regulating the expression of several fate-specifying transcription factors. However, the regulation of *lz*, and the control of *lz *expression levels in different cell types is not fully understood.

**Results:**

Here, we show a genetic interaction between Tramtrack69 (Ttk69) a key transcriptional repressor and an inhibitor of neuronal fate specification, and *lz*, the master patterning gene of cells posterior to the morphogenetic furrow in the *Drosophila *eye disc. Loss of Ttk69 expression causes the development of ectopic R7 cells in the third instar eye disc, with these cells being dependent upon Lz for their development. Using the binary UAS Gal4 system, we show that overexpression of Ttk69 causes the loss of *lz*-dependent differentiating cells, and a down-regulation of Lz expression in the developing eye. The loss of *lz*-dependent cells can be rescued by overexpressing *lz *via a GMR-*lz *transgene. We provide additional data showing that factors functioning upstream of Ttk69 in eye development regulate *lz *in a Ttk69-dependent manner.

**Conclusions:**

Our results lead us to conclude that Ttk69 can either directly or indirectly repress *lz *gene expression to prevent the premature development of R7 precursor cells in the developing eye of *Drosophila*. We therefore define a mechanism for the tight regulatory control of the master pre-patterning gene, *lz*, in early *Drosophila *eye development and provide insight into how differential levels of *lz *expression can be achieved to effect specific cell fate outcomes.

## Background

Eukaryotic cellular tissues are generally comprised of several cell types, many of which may be derived from a common pool of precursor cells. How such developmentally equivalent cells become distinct from one another remains a fundamental question in developmental biology. Specification of cell fates involves the interpretation of multiple signalling pathways by individual cells. The developing eye of *Drosophila *has been extensively used as a model system to determine how common signalling pathways can induce the generation of cellular diversity. In particular, specification of the R7 photoreceptor cell fate has been a principal paradigm for elucidation of how cell fates are established in response to signalling cues [1].

The adult *Drosophila *eye is comprised of approximately 800 ommatidia, with each ommatidium containing eight photoreceptor neurons surrounded by a collection of non-neuronal support cells [2]. The eye begins its development from the eye imaginal disc epithelium during mid-third larval instar, with the morphogenetic furrow progressing anteriorly across the disc, marking the onset of cell differentiation and pattern formation [3]. Photoreceptor R8 is the first cell established in the eye, its recruitment mediated by signalling events coordinated by the furrow [3,4]. Three pairs of photoreceptors, R2/5, R3/4 and R1/6, are then subsequently recruited to each ommatidial cluster, their recruitment being dependent upon reiterative induction of the epidermal growth factor receptor (EGFR) signalling pathway [5-8]. The last photoreceptor to be recruited is the R7 cell. Addition of non-neuronal lens secreting cone cells, supporting pigment cells, and the generation of sensory bristle cells make up the full complement of ommatidial cells [4,9].

Induction of the R7 cell has been the most extensively studied cell differentiation event in the eye. With respect to common signalling events, the Notch (N) signalling pathway and the receptor tyrosine kinases (RTKs), EGFR and Sevenless (Sev), have been shown to be necessary for induction of the R7 fate [10-15]. Loss of N signalling has been shown to cause the R7 precursor cell to adopt an R1/R6 cell fate. Conversely, ectopic N activation in R1/6 cells is sufficient to covert these cells into R7 cells [11,15]. The ability of N to potentiate R7 development is dependent on the expression of the N ligand, Delta, in R1/6 photoreceptors [11,15]. Moreover, N may induce R7 fate differentiation in the presumptive R7 cell by both activating R7-cell-specific determinants, and repressing R8 cell determinants [11,14,15]. Successive episodes of EGFR activation of the Ras/MAPK (Mitogen-Activated Protein Kinase) signalling cascade has been shown to be a requirement for recruitment of all photoreceptor neurons to the ommatidium, including the R7 cell [5,6,8]. In contrast, Sev signalling is restricted to the presumptive R7 cell, with loss of Sev signalling specifically resulting in the trans-determination of the presumptive R7 cell into a non-neuronal cone cell [10,12,13]. While Sev and EGFR both feed into the same signal transduction pathway, high levels of RTK activation in the presumptive R7 cell may be required to overcome repressive mechanisms specific to the R7 cell itself (reviewed in [1]). In the presumptive R7 cell, high levels of RTK signalling result in the expression of a novel nuclear gene *phyllopod *(*phyl*) [16,17]. Phyl functions as an adaptor protein in the R7 nucleus, recruiting the neuronal inhibitor Tramtrack (Ttk) into a complex with Seven in absentia (Sina) and Ebi [18-21]. Ttk RNA is alternatively spliced, giving rise to two zinc-finger DNA binding proteins, Ttk69 and Ttk88. Both Ttk isoforms share a common N-terminal region containing a BTB/POZ (Broad Complex Tramtrack Bric-a-Brac/Pox virus and Zinc finger) domain, but have alternative sets of Cys_2_-His_2 _zinc-fingers in the carboxyl fragment, resulting in the two isoforms having different DNA-binding specificities [22]. Both Ttk isoforms function to block neuronal fate specification in the third instar developing eye disc, and are presumed to be antagonists of RTK signalling, since overexpression of either isoform results in a failure of photoreceptor recruitment [21-24]. The recruitment of Ttk88 into the Sina-Ebi complex in R7 precursor cells leads to Ttk88 ubiquitination and post-translational degradation by proteolysis [20]. Targeted degradation of Ttk88 relieves the inhibition of the neuronal cell fate, allowing R7 fate specification to proceed. Evidence suggests that Ttk69 is also targeted for degradation through induction of RTK signalling [19,25,26]. Additional studies have also implicated the RNA-binding protein Musashi (Msi) in the translational inhibition of Ttk69 in R1/6/7 precursor cells in the developing eye [27,28]. While Ttk needs to be degraded for R7 fate specification to proceed, its presence is required for the formation of non-neuronal cone and pigment cells, highlighting the importance for tight regulatory control of cell-specific transcription factors in order to correctly specify cell fate within the developing eye disc [19,26,29].

The R7 cell arises from a population of undifferentiated cells surrounding the already recruited five-cell neuronal pre-cluster of R8/2/5/3/4 cells. These unspecified cells have undergone a second round of mitosis, an event required for re-population of the epithelium for the additional recruitment of the remaining photoreceptors, and non-neuronal supporting cells. The recruitment and differentiation of cells arising from the second mitotic wave requires the combinatorial inputs of N, EGFR and Sev signalling [30-33]. Additionally, these cells require the expression of Lozenge (Lz), a member of the RUNX family of transcription factors [34]. RUNX proteins are critical in development, since loss of RUNX protein function can lead to stomach cancer [35], the development of acute myeloid leukemia [36] or other severe developmental defects [37].

In its role in eye development, Lz has been described as a pre-patterning factor, since its expression within a pool of equipotent undifferentiated cells is required for the subsequent recruitment and differentiation of R1/6 and R7 cells, cone and pigment cells [30,38]. In the absence of Lz function, these cell types fail to correctly differentiate and excessive apoptosis in third-instar eye discs can be observed [38-42]. Lz, in combination with other factors, is required to regulate a number of cell specific transcription factors expressed posterior to the second mitotic wave. For instance, Lz acts in a combinatorial manner with Yan, PointedP2 and Suppressor of Hairless (Su(H)) to restrict *D-Pax2/shaven *expression to the cone cell precursors in third instar eye discs [30,43]. The regulation of *prospero (pros) *expression in R7 and cone cells is also dependent upon a combination of upstream transcription factors, including Lz, Pointed and Yan [32,41]. Lz has also been shown to negatively regulate *seven-up *in R7 and cone cells [34,40], *deadpan *(*dpn*) expression in cone cells [43], and *Bar *expression in R1 and R6 cells [34,40].

Although studies have demonstrated the importance of Lz in the specification of differentiated cell types in the eye disc after the second mitotic wave, *lz *gene regulation itself is not fully understood. While *lz *expression is initially activated in undifferentiated cells by Sine Oculis (So) and Glass (Gl) [33], evidence suggests that *lz *expression levels are up-regulated in differentiating cell types [39,41]. There is also some evidence to suggest that the Ras1/MAPK signalling pathway blocks the up-regulation of *lz *expression in undifferentiated cells through repression by the Yan protein [39,41].

Additional complexity to the regulation of *lz *gene expression is added by the findings that *lz *mRNA is alternatively spliced during eye development, producing a full length isoform (826aa; c3.5) and an isoform lacking exon V (705aa; Δ5 [41]). Exon V encodes a conserved ETS interaction domain, and yeast two hybrid screens showed the direct interaction between Lz and the ETS factor PointedP2, with this interaction removed upon site directed mutagenesis of ETS interacting sequences within this exon [41]. Exon V is critical for the development of presumptive R7 cells in the third instar eye disc, since R7 precursor cells can still develop in a severely truncated *lz *mutant with this exon intact.

In this study, we show that R7 development in third instar eye discs is dependent upon Lz function, and that Ttk69 may play a role in the direct or indirect repression of *lz *gene expression in cell types competent to develop as R7 cells. We show that loss of Ttk69 function results in the development of ectopic R7 cells in third instar eye development. These ectopic R7 cells are dependent upon Lz function for their development. Conversely, overexpression of Ttk69 results in loss of all *lz*-dependant differentiated cell types in the developing eye disc along with a marked decrease in Lz expression in both undifferentiated and differentiated cell types. Interestingly, by re-introducing Lz into a developing eye where Ttk69 is over-expressed, we could partially rescue cells expressing the *lz*-dependent factors Bar, Pros and Cut. We could also partially rescue the expression of the R7-cell-specific marker Klingon. Together, these results suggest that the loss of cells in the Ttk69 over-expressing lines was largely due to the removal of Lz function.

Additionally, we show that Sina and Msi, factors upstream of Ttk69 in eye development, play a Ttk-dependent role in *lz *gene regulation in R7 precursor cells. The elucidation of the function of Ttk69 in *lz *gene repression in a subset of cells provides a possible mechanism for the tight control of *lz *expression required for the correct differentiation of cell types during early eye development.

## Results

### *lozenge *mutations suppress *tramtrack *loss of function eye phenotypes

**Figure 1:**
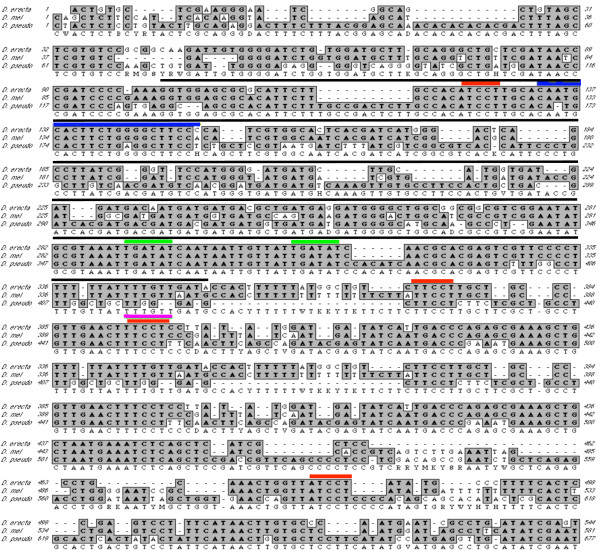
**Nucleotide alignment of a conserved region of the *lz *eye enhancer between *D. erecta*, *D. melanogaster *(*D.mel*) and *D. pseudoobscura *(*D. pseudo*)**. Highly similar sequences to the *D. melanogaster lz *eye enhancer region were identified in *D. erecta *and *D. pseudoobscura*. In this region, four putative Ttk69 core binding sites (red; AGGA-like sequences) were conserved across three species. Binding sites for Sine oculis (green) and Glass (blue), which are known regulators of *lz *gene expression, are also highly conserved. A conserved ETS binding site (pink) overlapping a Ttk69 core site is shown. A 251 bp minimal enhancer region which is essential for *lz *expression in undifferentiated cells is depicted (black line). Conserved nucleotides are shaded grey.

Lz has been shown to regulate a number of cell specific transcription factors expressed after the second mitotic wave in the developing eye [34,38,40] however, the regulation of *lz *itself is not fully understood. *lz *expression is initially activated in undifferentiated cells by So and Gl [33], and evidence also suggest that the ETS factor Yan prevents up-regulation of *lz *expression in undifferentiated cells [39,41]. So, Gl and Yan binding sites are all present in the *lz *eye-specific enhancer region, a critical functional region required for the correct expression of *lz *in the developing eye, located in the second intron of the *lz *gene (fig. 1; [33,38]). We conducted a multiple sequence comparison of this region across three *Drosophila *species, *D. melanogaster*, *D. pseudoobscura *and *D. erecta*, and found that binding sites for Gl [33,44], So [33,45] and the ETS factors Yan and PointedP2 [46] are conserved across these *Drosophila *species (fig. 1). Two other ETS binding sites have been identified in a second region of the eye enhancer and have been previously shown to be conserved in *D. simulans*, *D. melanogaster *and *D. erecta *[39]. Additionally, we identified four conserved putative Ttk69 core binding sites (fig. 1; [25,46]), suggesting that Ttk69 is a likely candidate to regulate *lz *gene expression in *Drosophila *eye development.

**Figure 2:**
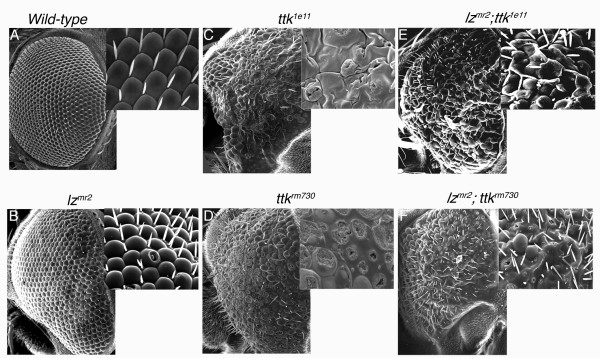
***ttk *loss of function phenotypes are suppressed in a *lz*^*mr*2 ^mutant background**. **A**. Scanning Electron Microscopy (SEM) of a wild-type (wt) adult eye. **B**. SEM shows that the *lz*^*mr*2 ^mutation results in a mild eye phenotype, with ommatidial disorganisation observed at the posterior rim of the adult eye. Insets represent higher magnification images in this and subsequent panels, and posterior is to the right in all images. **C**. SEM of *ttk*^1*e*11 ^mutant adult retina (loss of Ttk69; tissue generated using the Flp/FRT clonal system) shows severe retinal degeneration, with severely disrupted ommatidia. Severe scarring across the retina and failure of bristle formation is shown. Micrographs were taken from flies where clones were generated across nearly all the eye (*w*^- ^tissue; not shown). **E**. When *ttk*^1*e*11 ^mutant clones were generated in a *lz*^*mr*2 ^background, the *ttk*^1*e*11 ^mutant phenotype was partially suppressed, with the degree of ommatidial scarring reduced, and bristle formation restored. **D**. The eye phenotype caused by the generation of *ttk*^*rm*730 ^mutant tissue (loss of Ttk69 and ttk88) across the majority of the eye results in a severely deformed ommatidia and a lack of bristle development. **F**. The *ttk*^*rm*730 ^eye phenotype can be partially rescued when clones are generated in a *lz*^*mr*2 ^mutant background, with the degree of ommatidial scarring reduced, and the restoration of ommatidial structure and bristle formation observed.

To explore the genetic relationship between Ttk and Lz, the adult eye was used to investigate phenotypic interactions between *lz *mutants and mutations in candidate regulatory genes. *ttk *loss of function mutant clones were generated in a *lz*^*mr*2 ^hemizygote male mutant background, and the resultant eye phenotype was examined for phenotypic enhancement or suppression. The *lz*^*mr*2 ^mutation is caused by a P-element insertion in the 5' untranslated region of the gene [41] and adult males of this genotype exhibit minor ommatidial defects at the posterior rim of the eye, making this mutant amenable for a genetic interaction screen (fig. 2B). Using an allele known to result in the specific loss of Ttk69 but not Ttk88 (*ttk*^1*e*11^; [26]), and an allele known to result in the loss of both Ttk69 and Ttk88 (*ttk*^*rm*730^; [26]), *ttk*^- ^clones were generated by mitotic recombination using the FLP/FRT system [47] with the Flp recombinase placed under the control of the *eyeless *eye enhancer [48]. Additionally, a cell lethal mutation (3R3.7) was also introduced into the system, resulting in the death of the twin-spot cells homozygous for the 3R3.7 mutation, but not the *ttk*^- ^mutation. This leaves only *ttk*^- ^homozygous cells and non-recombinant heterozygous cells. As a result, *ttk*^- ^clones occupied the majority of the eye [48]. Scanning electron microscopy revealed that loss of Ttk69 function, or both Ttk69 and Ttk88, in the eye resulted in severe degeneration of the corneal lens, with ommatidia and sensory bristles failing to properly develop (fig. 2C-D). These results were consistent with those previously described [26]. Following the generation of large patches of *ttk*^*rm*730 ^or *ttk*^1*e*11 ^mutant clones in *lz*^*mr*2 ^hemizygote males, we observed the rescue of *ttk*^- ^mutant eye phenotypes (fig. 2E-F). In *lz*^*mr*2^*;ttk*^- ^double mutant tissue, ommatidial structure was present and the eye appeared less scarred. Furthermore, sensory bristles developed in the double mutants (fig. 2E-F), whereas they were absent in the *ttk*^- ^mutant tissue (fig. 2C-D). These results show that *lz *mutations can partially suppress the severe eye phenotypes of *ttk *loss of function mutants, suggesting that Lz functions downstream of, or in parallel to, Ttk in the developing eye.

### Loss of Ttk69 function alone is sufficient to cause development of ectopic presumptive R7 cells early in development

Loss of Ttk88 function has been shown to result in the development of ectopic R7 cells in the adult retina [23,24]. However, another study analysed expression of the neuronal marker Elav in *ttk*^1*e*11 ^and *ttk*^*rm*730 ^mutant clones earlier in development and found that ectopic photoreceptors never developed in third instar larval ommatidial clones [26]. We therefore investigated whether ectopic presumptive R7 cells could be detected in *ttk*^1*e*11 ^and *ttk*^*rm*730 ^mutant clones generated by the *eyFlp/FRT *method in third instar eye discs by using alternative R7 cell markers.

**Figure 3:**
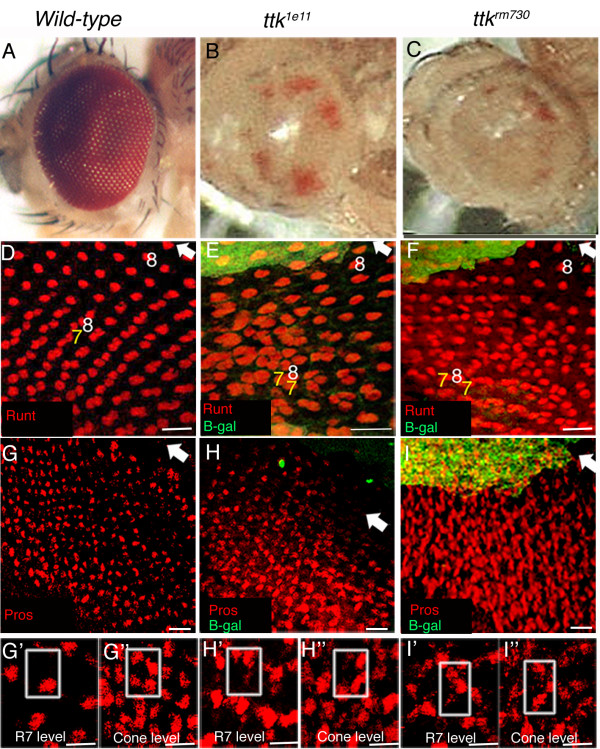
**Ectopic Runt and Prospero labelled R7 cells develop in *ttk*^1*e*11 ^and *ttk*^*rm*730 ^mutant ommatidia**. **A**. Light micrographs of a wild-type (wt) eye and **(B-C) ***ttk*^1*e*11 ^and *ttk*^*rm*730 ^mutants show *w*^- ^clonal tissue covering most of the eye. **D-F**. Projected confocal images of third instar eye discs labelled with Runt (Red) and β-galactosidase (β-gal; green). Arrow marks the morphogenetic furrow (Mf); posterior is towards the bottom. Absence of β-gal represents *ttk*^1*e*11 ^**(E) **and *ttk*^*rm*730 ^**(F) **clonal cells. In wt discs **(D)**, one Runt labelled R8 cell (8) is observed in each ommatidia for six rows posterior to the Mf. At row seven, one R7 cell (7) is recruited. In both *ttk*^1*e*11 ^**(E) **and *ttk*^*rm*730 ^**(F) **discs, ectopic Runt positive R7 cells appear seven rows posterior to the Mf. **G-I**. Projected confocal images of Pros labelled cells (red) reveal an increase in Pros expression in *ttk*^- ^clonal tissue **(H-I) **compared to wt tissue **(G)**. Single planar images **(G'-I") **show the presence of one Pros positive cell in the R7 cell plane of wt eye discs **(G')**, while four Pros labelled cone cells (CCs) are present in a more apical plane **(G")**. In *ttk*^1*e*11 ^and *ttk*^*rm*730 ^ommatidia, more than one Pros labelled cell is often observed in the R7 cell plane **(H', I')**, and less than four Pros labelled CCs can be observed in the apical plane **(H", I")**. The boxed region in each panel represents one ommatidium. Scale Bars in D-I indicate 10 μm, in G'-I", 5 μm.

Runt protein expression can normally be detected in R7 and R8 cells in the developing eye [39,49,50]. A projected confocal image of Runt labelled wild-type eye discs show a single R8 cell per ommatidia just posterior to the furrow (fig. 3D). Seven rows posterior to the furrow, when R7 cells are normally recruited to ommatidial clusters, Runt positive R7 and R8 cells can be observed (fig. 3D). Confocal imaging of Runt labelled *ttk *mutant discs showed that R8 cells are recruited normally in both *ttk*^1*e*11 ^and *ttk*^*rm*730 ^mutant clones. However, ectopic R7 cells were observed from approximately the seventh row posterior to the furrow in both *ttk*^1*e*11 ^and *ttk*^*rm*730 ^mutant ommatidia (fig. 3E-F). We found that 87.1% (N = 350) of *ttk*^1*e*11 ^mutant ommatidia contained between 2-3 Runt-labelled presumptive R7 cells in the apical R7 focal plane approximately seven rows posterior to the furrow in the disc epithelium, while 89.8% of *ttk*^*rm*730 ^mutant ommatidia also exhibited this phenotype (N = 433). *ttk*^1*e*11 ^and *ttk*^*rm*730 ^clones were easily characterised by a lack of β-galactosidase staining in the early eye disc, and mutant clones constituted the majority of the eye disc, a result consistent with the large areas of *w*^- ^clonal patches observed in the adult eye (fig. 3B-C).

To further confirm the identity of these ectopic Runt labelled cells in *ttk*^- ^mutant ommatidia, analysis of *ttk *loss of function mutant clones with the presumptive R7 cell Pros was also undertaken. Pros protein expression can normally be detected in R7 and cone cells in the third instar developing eye epithelium [41,49,51]. Pros is expressed at higher levels in the R7 precursor cell than the cone cells at this stage [32,41], with these cell types being located at distinct depths (focal planes) in the developing eye disc, the cone cells being the most apical [52]. Confocal imaging showed the presence of ectopic Pros labelled presumptive R7 cells in both *ttk*^*rm*730 ^and *ttk*^1*e*11 ^mutant ommatidia (fig. 3H, I). High magnification images of the mutant disc showed the presence of at least two labelled cells in some ommatidia in the R7 cell focal plane (fig. 3H', I'), compared to a single cell in the same plane of a wild-type disc (fig. 3G'). Normally, the four Pros labelled cone cells are evident in a focal plane above R7 (fig. 3G'') as originally described by Tomlinson and Ready [52]. Pros labelled cone cells were also present in *ttk*^- ^mutant clones (fig. 3H''-I''). However, less than the normal complement of cone cells was often observed in both *ttk*^1*e*11 ^and *ttk*^*rm*730 ^mutant ommatidia, suggesting a trans-determination of non-neuronal cone cells into presumptive R7 cells in the absence of Ttk function. Taken together, both Runt and Pros labelling of *ttk*^1*e*11 ^and *ttk*^*rm*730 ^mutant eye discs showed the presence of ectopic presumptive R7 cells in the early developing eye of mutant ommatidia lacking Ttk69 function alone, or lacking both Ttk69 and Ttk88 function.

Since ectopic presumptive R7 cells were observed in the larval disc epithelia of *ttk*^- ^mutant clones, we next analysed whether these ectopic R7 cells also expressed *lz*. Therefore, *ttk*^*rm*730 ^and *ttk*^1*e*11 ^clones were generated in a *lz *enhancer trap line (*lz*^*Gal*4^) background. *lz*^*Gal*4 ^flies contain a P-GawB insertion in the 5' untranslated region of the gene and thus the yeast Gal4 transcriptional activator is expressed under the control of *lz *enhancer elements [39-41]. The eye phenotype of *lz*^*Gal*4 ^*UAS-GFP *flies is normal, and the enhancer trap has been shown to faithfully report the expression of *lz *in the developing eye, with the expression pattern comparable to Lz protein expression [38,40].

**Figure 4:**
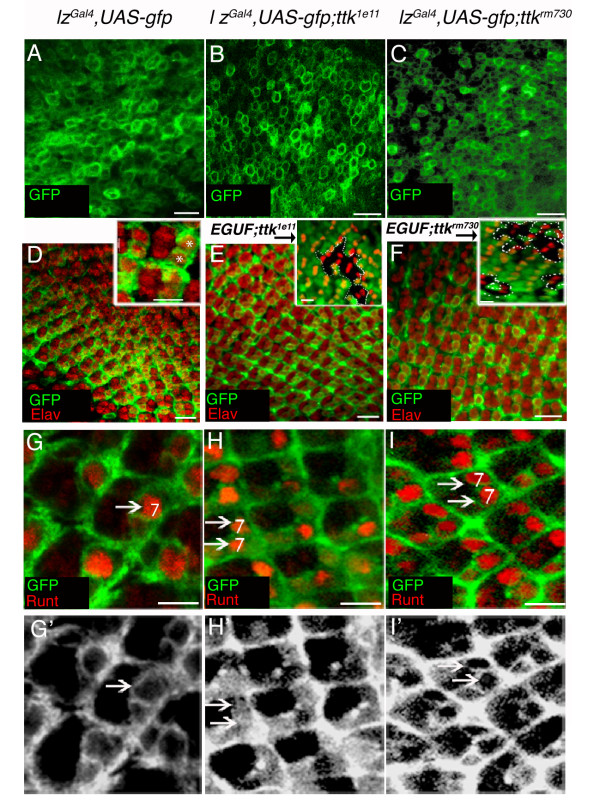
**Ectopic *lz *expressing R7 cells are observed in *ttk*^1*e*11 ^and *ttk*^*rm*730 ^mutant ommatidia**. **A**. A single planar confocal image of undifferentiated cells shows cytoplasmically localised *lz-*driven GFP expression (green) in a disc from a *lz*^*Gal*4^, *UAS-gfp *fly. **B-C**. No change in GFP expression could be detected in *ttk*^- ^mutant undifferentiated cells generated in a *lz*^*Gal*4^, *UAS-gfp *background. For B-C, E-F (excluding insets), H, H', I and I', images were taken from regions of the disc with large *ttk*^- ^clonal patches (β-gal in far red not shown). **D-F**. Confocal images of Elav labelled neuronal cells (red) and *lz*-driven GFP expressing cells (green) reveal that *ttk*^1*e*11 ^**(E) **and *ttk*^*rm*730 ^**(F) **mutant R1 and R6 cells develop normally. **D **(inset) shows a higher magnification image of wt, gfp-expressing R1 and R6 cells co-expressing Elav. **E **and **F **insets show mosaic discs of *ttk*^- ^clones (GFP negative; outlined) stained with Bar (red) and GFP (green). No loss of, or extra, Bar-expressing cells were observed. **G-I**. Confocal images show ectopic GFP-expressing R7 cells co-expressing Runt (red) in *ttk*^- ^mutant discs **(H-I)**. **G'-I'**. *lz*-driven GFP in the R7 cell plane is in grayscale. Scale Bars in A-F indicate 10 μm, In D-F (insets) and in G-I' they indicate 5 μm.

*lz*-driven GFP expression is normally detected in the cytoplasm of undifferentiated cells posterior to the morphogenetic furrow (fig. 4A; [40]). Analysis of *lz*-driven GFP expression in *ttk*^1*e*11 ^and *ttk*^*rm*730 ^mutant undifferentiated cells revealed no visible alteration to the levels of GFP expression, or the numbers of GFP-expressing cells (fig. 4B-C). As cells begin their differentiation process, *lz*-driven GFP expression can be detected in R1,6 and R7 photoreceptors (fig. 4D, G; [38,40]), and in cone cells (not shown; [38,40]). In both *ttk*^1*e*11 ^and *ttk*^*rm*730 ^mutant clones, photoreceptors R1 and R6 cells developed normally and also expressed *lz*-driven GFP (fig. 4E-F). R1 and R6 cells were identified by the co-localisation of *lz*-driven GFP with the neuronal marker Elav, and by their position in the ommatidia. Additionally, Bar expression was analysed in *ttk*^1*e*11 ^and *ttk*^*rm*730 ^clonal patches in an Eyeless Gal4;UAS-Flp;FRT82B-UbiGFP (EGUF) background (fig. 4E-F insets). Bar specifically labels R1 and R6 cells in the developing eye [53]. In all negatively marked *ttk*^- ^mutant clonal patches analysed, no loss of Bar expressing cells, or ectopic Bar expression, was ever observed. Taken together, the early development of presumptive R1 and R6 cells in third instar disc epithelia is not perturbed by the loss of Ttk69 function.

Analysis of *ttk*^1*e*11 ^and *ttk*^*rm*730 ^mutant ommatidia with the presumptive R7 cell marker Runt showed that ectopic R7 cells present in the mutant disc epithelia also expressed *lz*-driven GFP (fig. 4H-H', I-I'). Additional labelling of mutant ommatidial clones with Pros revealed the presence of more than one *lz*-expressing R7 cell in the R7 cell focal plane (not shown). GFP-expressing cone cells do develop in *ttk*^- ^ommatidia, although these cells are disorganised and often the incorrect complement of cone cells (less than four) was observed (not shown). These results reveal that the removal of Ttk69 function alone is sufficient to induce the development of ectopic R7 cells in the developing eye which also continue to express *lz*. Interestingly, while the presumptive R7 markers Runt and Pros clearly show the development of ectopic R7 cells in eye discs lacking Ttk69 function, analysis with the neuronal marker Elav did not reveal the presence of ectopic Elav-expressing cells in *ttk*^- ^mutant ommatidia (fig. 4E-F and not shown). This suggests that the ectopic presumptive R7 cells are arising from cells of the non-neuronal cell lineage, which is consistent with previously published reports [29].

### The development of ectopic R7 cells in *ttk*^1*e*11 ^mutants is dependent upon Lz function

**Figure 5:**
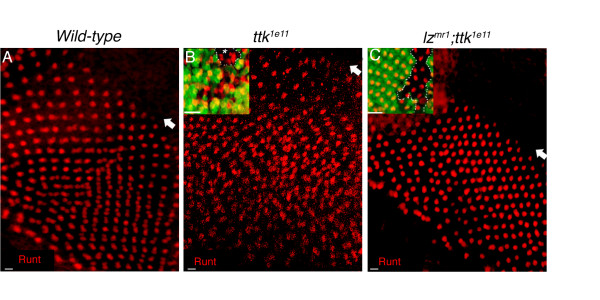
**R7 cell development in *ttk*^- ^mutant ommatidia is dependent upon Lz function**. **A**. Projected confocal image of Runt labelled cells in a wild-type disc show one R8 cell per ommatidial cluster for approximately six rows posterior to the morphogenetic furrow (Mf; arrow). At row seven, Runt-expressing R7 cells are recruited to each ommatidium and Runt "doublets" are observed. **B**. In the *ttk*^1*e*11 ^mutant ommatidia, ectopic Runt positive R7 cells are observed. **C**. Only single R8 cells are observed in developing ommatidia of *lz*^*mr*1^; *ttk*^1*e*11 ^ommatidia. **B **and **C **show discs where *ttk*^- ^clones occupied the majority of the eye disc (β-galactosidase not shown). **B **and **C **(insets) show mosaic images, where ectopic Runt positive cells can be observed in *ttk*^1*e*11 ^clonal patches (B inset; GFP negative clones outlined). **C Inset**. In *lz*^*mr*1 ^mutant discs, R7 cells are not recruited to the ommatidia, and the generation of *ttk*^1*e*11 ^clones in this background (outlined in inset) does not result in the recruitment of R7 precursor cells. Scale bars indicate 10 μm.

We next set out to determine whether Lz function is essential for the development of ectopic R7 cells in mutants lacking Ttk69 function. To address this, *ttk*^1*e*11 ^clones were generated in a *lz*^*mr*1 ^loss of function mutant background and the recruitment of R7 precursor cells was monitored using the Runt antibody. The *lz*^*mr*1 ^mutation is caused by a lesion in the eye specific enhancer region of the *lz *gene, and the levels of *lz *transcript produced in this mutant are at less than 10% of those produced in wild-type flies [40,42], resulting in the absence of the development of *lz*-dependent cell types. Results showed that after the removal of Lz function in the absence of Ttk69 function, R7 cells failed to develop in the early developing eye (fig. 5A-C). These results indicate the Lz is absolutely required for R7 cell development and suggest that Lz may function downstream of Ttk69 in this context.

### Overexpression of Ttk69 results in the down-regulation of *lz *and the subsequent loss of *lz*-dependent cell types in the developing eye

Our results have demonstrated that removal of Ttk69 function results in the development of ectopic R7 cells at the third instar developmental stage. These ectopic cells express *lz*-driven GFP, and are also dependent upon Lz function for their development. It is possible that Ttk69 may function to regulate *lz *expression levels in early eye development to prevent the ectopic development of R7 cells. Alternatively, these ectopic R7 cells may simply continue to express Lz because they are derived from the R7 equivalence group. We therefore examined whether Ttk69 has the ability to repress *lz *gene expression in the developing eye. First we over-expressed Ttk69 in *lz*-expressing cells by crossing *lz*^*Gal*4 ^into a *UAS-ttk69 *background and analysed the expression of *lz*-driven GFP along with the expression of specific presumptive cell fate markers. *lz*^*Gal*4^*;UAS-ttk69 *adult eyes exhibited severely disrupted eyes that lacked ommatidial structure and also exhibited severe scarring (not shown). This adult phenotype alone suggests that overexpression of Ttk69 in *lz*-expressing cells is enough to prevent the correct development of these cells.

**Figure 6:**
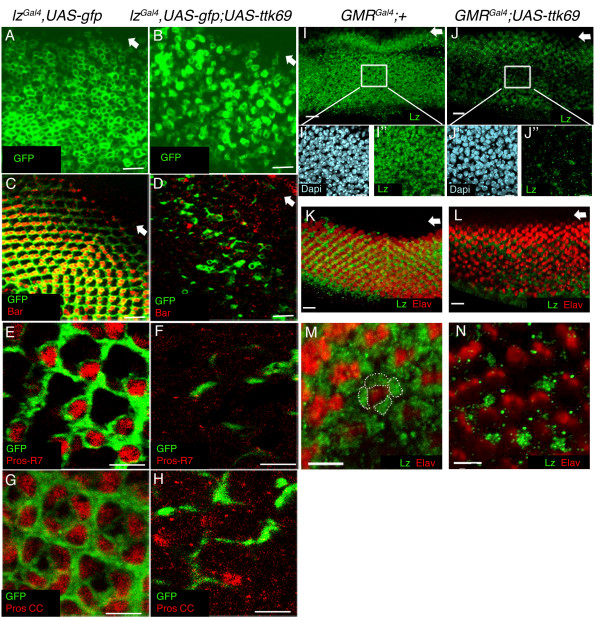
***lz*-driven GFP, and Lz protein expression are downregulated in cells overexpressing Ttk69**. **A-B**. Confocal images of GFP-expressing (green) undifferentiated cells in *lz*^Gal4^, UAS-gfp and *lz*^Gal4^, UAS-gfp;UAS-*ttk69 *eye discs. **C**. R1/6 cells in a *lz*^Gal4^, UAS-gfp disc co-stained with Bar (red) and GFP (green). **D**. In *lz*^Gal4^, UAS-gfp;UAS-*ttk69 *eye discs, Bar and GFP expression are downregulated. **E**. A confocal image of a *lz*^Gal4^, UAS-gfp disc shows a Pros-expressing R7 cell (red) co-expressing GFP in each ommatidium. **F**. R7 cells are lost upon Ttk69 overexpression. **G**. Image of the cone cell (CC) plane in *lz*^Gal4^, UAS-gfp discs shows four Pros-labelled (red), GFP-expressing (green) CCs per ommatidium. **H**. Most CCs are lost upon Ttk69 overexpression. **I**. Projected image of Lz antibody (green) staining in undifferentiated cell planes (non Elav-expressing) of GMR^Gal4^;+ eye discs. **J**. Down-regulation of Lz (green) is observed upon Ttk69 overexpression. **I'-I"**. Most dapi-stained undifferentiated cells (light blue; **I'**) in control discs also express Lz (**I"**). Numerous dapi stained undifferentiated cells were observed in GMR^Gal4^;UAS-*ttk69 *eye discs (**J'**), but few expressed Lz (**J"**). **K**. Lz is expressed in R1, 6 and R7 cells of control GMR^Gal4^;+ eye discs (Elav, red; Lz, green). **L**. Co-localisation of Elav and Lz was not observed in GMR^Gal4^;UAS-*ttk69 *eye discs, and a decrease in Lz expression was observed. **M**. Imaging in the apical plane of control discs show four Lz-labelled CCs (outlined). **N **Few Lz labelled CCs are observed in GMR^Gal4^;UAS-*ttk69 *eye discs. Scale bars: A-D and I- L all indicate 10 μm, E -H, I'-J", M and N indicate 5 μm.

Further analysis of *lz*-driven GFP in disc epithelia of *lz*^*Gal*4^, *UAS-GFP;UAS-ttk69 *third instar larvae was undertaken. Single planar confocal images of undifferentiated cells in the *lz*^*Gal*4^, *UAS-GFP;UAS-ttk69 *mutant disc epithelia showed that although less cells were present in this focal plane than in control discs (fig. 6A-B), the levels of *lz-*driven GFP expression did not appear to be reduced upon overexpression of Ttk69.

In neuronal and non-neuronal differentiating cells, specific antibodies were used in conjunction with the *lz *enhancer trap line to mark each cell type. Photoreceptors R1 and R6 were identified using an antibody against the Bar antigen ([40,53,54]; fig. 6C). Our results showed that Bar expression was lost in the *lz*^*Gal*^, *UAS-GFP;UAS-ttk69 *disc epithelium (fig. 6D), and *lz*-driven GFP expression was severely depleted in the R1 and R6 cell plane.

To analyse R7 and cone cell development in the *lz*^*Gal*^, *UAS-GFP;UAS-ttk69 *third instar disc epithelium, Pros was used in conjuction with the *lz *enhancer trap line to specifically mark these cells (fig. 6E, G). In the Ttk69 overexpression line, high magnification confocal imaging revealed few Pros and *lz*-driven GFP labelled R7 and cone cells in the developing third instar eye discs (fig. 6F, H). The depletion of other cell specific markers such as Runt in R7 cells and Cut in cone cells was also observed (data not shown). Since both *pros *and *Bar *are, in part, regulated by Lz, these results support the hypothesis that Ttk69 can repress *lz *gene expression in the developing eye.

A caveat to the above described experiment is that the system utilises the *lz*^Gal4 ^enhancer to simultaneously monitor UAS-GFP expression and drive UAS-ttk69, when Ttk69, in turn, could then act to repress the *lz *enhancer element and no longer drive the UAS-*ttk69 *transgene. This experiment is therefore dependent upon the relative stability of Ttk69 in the third instar disc epithelium. While we did observe a perturbation to the development of differentiated *lz*-expressing cell types, it remains possible that *lz*-driven GFP expression was produced initially in undifferentiated cells before Ttk69 could be produced at high enough levels to repress *lz*. We therefore obtained a Lz antibody and examined Lz protein expression in discs where a Gal4 driver under the control of the *glass *multimer repeat (GMR) promoter was used to drive the UAS-*ttk69 *transgene in all cells posterior to the morphogenetic furrow in developing eye discs [55].

In control (*GMR*^*Gal*4^;+) eye discs, a projected confocal image of all cells in the basal undifferentiated cell plane reveal that Lz is expressed in most, if not all, undifferentiated cells posterior to the morphogenetic furrow (fig. 6I). Upon Ttk69 overexpression, a marked reduction in the number of Lz expressing cells, and the levels of Lz expression, in the undifferentiated cell plane was observed (fig. 6J). Interestingly, Lz appeared to be expressed in a number of undifferentiated cells just posterior to the furrow, then expression decreased in the middle and posterior portions of the disc. This could simply reflect a delay in the onset of Ttk69 overexpression by the GMR-Gal4 transgene. Alternatively, Ttk69 could repress *lz *expression in specific pools of undifferentiated precursor cells. High magnification images of the middle disc region revealed that most, if not all, undifferentiated cells in control discs stained with the DNA marker 4',6-diamidino-2-phenylindole (Dapi) also expressed Lz (fig. 6I'-I"). In contrast, overexpression of Ttk69 in undifferentiated cells did not cause a reduction in the number of Dapi-stained cells (fig. 6J'), but a clear reduction of Lz expression was observed (fig. 6J").

In the differentiated region of the control disc epithelia, Lz expression was observed in R1, R6, R7 and cone cells (fig. 6K, M). This is consistent with the published expression pattern of Lz [38], Additionally, we confirmed Lz antibody specificity by staining Lz null mutant disc epithelia with the antibody and observed no Lz expression (not shown). A reduction of Lz expression was observed in differentiated cells of disc epithelia where Ttk69 was over-expressed (fig. 6L, N). Very few cells expressing both Elav and Lz were ever observed in these developing eye discs, and few cone cells in the apical cell plane expressed Lz. Taken together, these results show that Ttk69 can repress *lz *expression in the developing eye.

### Loss of *lz*-dependent cells in Ttk69 overexpression flies can be rescued by expressing a GMR-*lz *transgene lacking *lz *regulatory regions

Our results have shown that Ttk69 overexpression leads to repression of *lz *expression, which in turn results in the loss of *lz*-dependent differentiating cell types in third instar eye disc epithelia. We next tested whether overexpression of Lz could rescue the loss of *lz*-dependent cells types observed from Ttk69 overexpression in developing eye discs. For this experiment, a *GMR-lz *transgene containing the full length *lz *3.5 kb cDNA under the control of the GMR promoter, but lacking the *lz *eye specific enhancer and upstream promoter regions [34], was crossed into the *GMR-Gal4;UAS-ttk69 *mutant background, and eye discs were analysed for the rescue of photoreceptors R1,6,7 and cone cells. By itself, the GMR-*lz c3.5 *transgene (referred to in subsequent text as *GMR-lz*) causes ectopic development of the presumptive R7 cell and a mild disorganisation of developing ommatidia (not shown), indicating that high levels of Lz expression can indeed promote the R7 cell fate early in *Drosophila *development.

**Figure 7:**
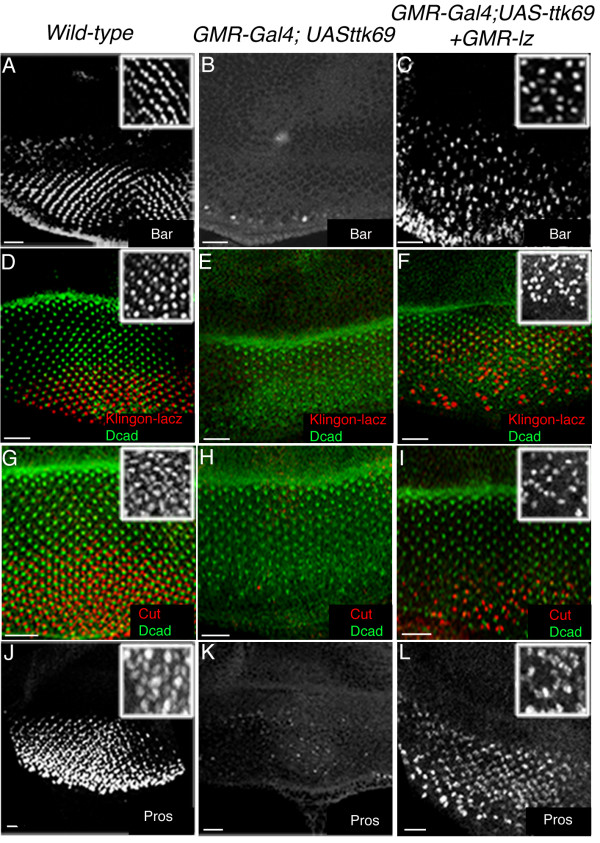
**The expression of GMR- *lz *can rescue cellular defects caused by the overexpression of Ttk69**. **A**. Imaging of Bar positive R1/R6 cells in wt eye discs. Inset in all panels shows higher magnification images. **B**. Bar expression is lost in GMR-Gal4; UAS-*ttk69 *discs. **C**. Partial rescue of R1/6 cells is shown in GMR-Gal4; UAS-*ttk69*/GMR-*lz *eye discs. **D**. Anti-β-galactosidase (β-gal) staining (red) of the Klingon-lacZ line (B38) shows high levels of *klingon *gene expression in R7 cells and lower expression levels in other cell types. **E**. B38 enhancer trap activity was only detected at background levels in GMR- Gal4; UAS-*ttk69 *eye discs. **F**. Partial rescue of β-gal positive R7 cells is observed in GMR-Gal4; UAS-*ttk69*/GMR-*lz *eye discs. D-ECadherin staining (green) in D-F, and G-I was used to mark the furrow. **G**. Confocal image showing anti-Cut staining (red) of cone cells (CCs) in wt discs. **H**. Few Cut labelled CCs (red) are detected in GMR-Gal4; UAS-*ttk69 *discs. **I**. Partial rescue of CCs is observed in GMR-Gal4; UAS-*ttk69*/GMR-*lz *eye discs. **J**. Imaging of Pros labelled wt eye discs. **K**. Pros expression is markedly reduced in GMR-Gal4; UAS-*ttk69 *eye discs. **L**. A dramatic rescue of Pros in R7 and CCs is observed in GMR-Gal4; UAS-*ttk69*/GMR-*lz *eye discs. Scale Bars indicate 20 μm.

*GMR-Gal4;UAS-ttk69 *adult eyes are severely perturbed, being devoid of any ommatidial structure (not shown). Furthermore, Elav labelling of third instar *GMR-Gal4;UAS-ttk69 *eye discs showed that most neuronal cells failed to differentiate (see fig. 6L), which is not surprising given that Ttk69 is a general repressor of the neuronal cell fate [24,29,56-58]. We first used the Bar antibody to determine whether *lz*-dependent R1 and R6 cells could be rescued in *GMR-Gal4;UAS-ttk69/GMR-lz *eye disc epithelia. In *GMR-Gal4;UAS-ttk69 *eye discs, few Bar labelled cells were observed, with only 7.1% (N = 280) of ommatidia exhibiting at least one Bar positive cell (fig. 7B). Overexpression of Lz in the absence of the eye specific enhancer could partially alleviate this defect, with 32% of ommatidia (N = 312) showing the presence of at least one Bar positive cell (fig. 7C and inset).

To determine whether R7 cells could be rescued in *GMR-Gal4;UAS-ttk69/GMR-lz*, the R7-specific enhancer trap line B38-LacZ was recombined with the *GMR-lz *transgene and crossed into the *GMR-Gal4;UAS-ttk69 *background. The B38-LacZ line is an enhancer trap insertion in *klingon *(a novel member of the *Drosophila *immunoglobulin superfamily that is required for R7 cell development [57]) and is strongly expressed in R7 cells but only weakly expressed in other surrounding photoreceptors ([57,58]; fig. 7D). The use of an anti-β-galactosidase antibody to detect enhancer trap activity showed the depletion of R7 cells in *GMR-Gal4;UAS-ttk69 *eye discs, with as few as 3.9% of ommatidia (N = 310) ever showing the presence of a β-galactosidase positive cell (fig. 7E). However, in *GMR -Gal4;UAS-ttk69/GMR-lz*, many more β-galactosidase positive R7 cells (69%; N = 362) were observed in the developing ommatidia (fig. 7F).

Non-neuronal cone cells can normally be detected with the Cut antibody ([59]; fig. 7G). While few Cut labelled cone cells were observed in *GMR-Gal4;UAS-ttk69 *developing eye discs (fig. 7H), a proportion of Cut labelled cone cells were rescued in *GMR-Gal4;UAS-ttk69/GMR-lz *discs (fig. 7I), although no ommatidia with the full complement of cone cells were ever observed. Due to the disorganisation of these cells, absolute numbers could not be obtained.

Because *pros *is positively regulated by Lz [32], we asked whether Pros expression could also be rescued in *GMR-Gal4;UAS-ttk69/GMR-lz *discs. Few Pros labelled cells were ever observed in *GMR-Gal4;UAS-ttk69 *eye discs (fig. 7K). However, in *GMR-Gal4;UAS-ttk69/GMR-lz *discs, a significant increase in Pros expression was observed, particularly in the R7 cell plane (fig. 7L). Taken together, these results strongly suggest that some of the cellular phenotypes observed upon Ttk69 overexpression in third instar developing eye disc are at least partially due to changes in *lz *expression, since the simultaneous overexpression of Lz and Ttk69 leads to rescue of R1,6,7 photoreceptors and non-neuronal cone cells, all of which are dependent upon Lz for fate specification. Furthermore, because the *GMR-lz *transgene lacks *lz *regulatory regions, these results suggest that the regulation of *lz *by Ttk69 could potentially occur via the *lz*-eye enhancer region.

### *sina *and *msi *function as negative regulators of Ttk in R1/6/7 cells in early eye development

The normal specification of the presumptive R7 cell is prevented in *sina *loss of function mutants due to failure of Ttk88, and presumably Ttk69, degradation [19,21,60]. However the expression pattern of Sina in R1,3,4,6 and R7 cells [61] initiated the investigation of the function of Sina in these other cell types. Genetic experiments have suggested that the RNA-binding protein Msi functions redundantly with Sina to down-regulate Ttk69 in R1 and R6 cells in larval eye development [27]. In their experiments, Hirota and colleagues showed the loss of R1, R6 and R7 photoreceptors in *sina msi *double loss of function mutants, while all cells were recruited properly in *msi *null mutant developing ommatidia [27]. Furthermore, 30% of retinal sections from adult ommatidia of *sina msi *double mutants in a *ttk*^*osn *^heterozygous mutant background, a mutation that disrupts both Ttk69 and Ttk88 protein expression, exhibited the correct number of photoreceptor neurons, consistent with the hypothesis of negative regulation of Ttk by both Sina and Msi.

**Figure 8:**
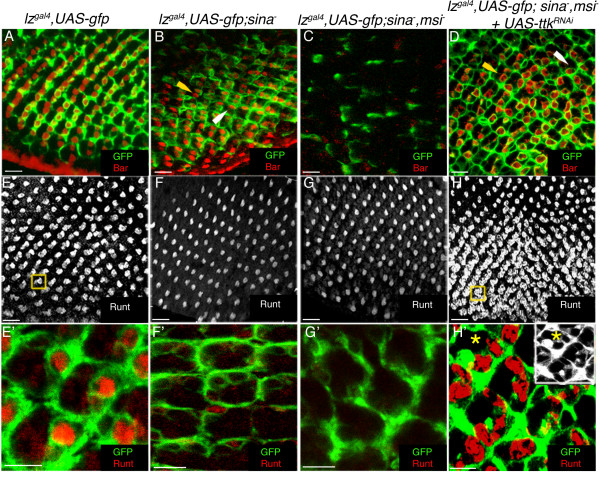
**Sina and Msi function as negative regulators of Ttk in R1/6/7 cells in early eye development**. **A**. Image of a *lz*^Gal4^, UAS-gfp disc stained with Bar (red) and GFP (green) **B**. In *sina*^2^/*sina*^3 ^mutants, R1/R6 cells develop (yellow arrowhead) and express *lz*-driven GFP (green). Occasionally, mutant ommatidia with only one Bar labelled cell can be observed (white arrowhead). **C**. In *lz*^Gal4^, UAS-GFP; *sina*^2^*msi*^1^/*sina*^3^*msi*^1 ^discs, Bar positive cells are lost and GFP expression is reduced. **D**. Knockdown of Ttk in a *sina*^2^*msi*^1^/*sina*^3^*msi*^1 ^mutant background by RNAi results in rescue of R1/R6 cells (yellow arrowhead). Occasionally, only one Bar positive cell is observed in the ommatidium of *lz*^Gal4^, UAS-GFP; UAS-*ttk*^*RNAi*^*;sina*^2^*msi*^1^/*sina*^3^*msi*^1 ^discs (white arrowhead). **E**. Image of Runt labelled R7/R8 cells (grayscale) in a *lz*^Gal4^, UAS-GFP disc (GFP not shown). The yellow box shows a "doublet" of R7/R8 cells in one ommatidium. **E'**. A higher magnification of Runt labelled R7 cells (red) expressing GFP (green). **F-G**. R7 cells are not observed in *lz*^Gal4^, UAS-GFP;*sina*^2^/*sina*^3 ^eye discs (**F-F'**), or in *lz*^Gal4^, UAS-GFP;*sina*^2^*msi*^1^/*sina*^3^*msi*^1 ^discs (**G-G'**). **H-H'**. In *lz*^Gal4^, UAS-GFP; UAS-*ttk*^*RNAi*^;*sina*^2^*msi*^1^/*sina*^3^*msi*^1 ^eye discs, ectopic R7 cells from approximately row 7 posterior to the furrow are observed (yellow box) (**H**), and these cells (red) express GFP (green; **H'**). The asterix shows two R7 cells in the same confocal plane. The inset shows GFP in grayscale. Scale Bars in A-H indicate 10 μm, in E'-H', 5 μm.

To further examine the relationship between Sina, Msi, Ttk69 and *lz *gene regulation, we examined *lz *gene expression in R1, R6 and R7 photoreceptor cells of *sina *loss of function mutant eye discs, and of *sina msi *double loss of function mutants using the *lz*^*Gal*4 ^enhancer trap line to drive GFP expression. Labelling of *sina *null mutant eye discs with the R1 and R6 photoreceptor marker Bar demonstrated that 87.2% of ommatidia contained two *lz*-expressing Bar positive cells (fig. 8B). However some ommatidia contained less than two Bar positive cells, indicating that Sina may play a minor role in the cell fate determination of R1 and R6 cells (fig. 8B). In *sina msi *double mutant eye discs very few *lz*-expressing Bar positive R1 and R6 cells were observed (1.2%, N = 250; fig. 8C). Analysis of R7-cell development in mutant eye discs with the presumptive R7 cell marker Runt showed that very few R7 cells are recruited to developing ommatitida in *sina *mutants (2.1%, N = 523; fig. 8F-F'), and in *sina msi *double mutants (.06%, N = 315; fig. 8G-G'). The possibility remains that fewer cells were recruited to differentiating ommatidia because fewer cells were available from the pool of undifferentiated cells.

To determine whether the mutant defects observed in *sina msi *double mutants could be attributed to failure of Ttk degradation in early eye development, we expressed a UAS-*ttk*^*RNAi *^line under the control of the *lz*^Gal4 ^driver in a *sina msi *double mutant background. Expression of the UAS-*ttk*^*RNAi *^transgene would result in the knockdown of both Ttk isoforms based upon the reported targeted Ttk sequence (not shown).

In *lz*^Gal4^, *UAS-gfp*; *UAS-ttk*^RNAi^; *sina*^-^*msi*^- ^eye discs, we observed a significant rescue of Bar and *lz*-expressing R1 and R6 cells (fig. 8D). At least one Bar positive cell was observed in 73.1% of ommatidia (N = 301), with the majority of ommatidia containing two Bar positive cells. The eye disc did appear disorganised and occasionally R1 and R6 photoreceptor cells were observed in an incorrect orientation (fig. 8D). Additionally, the recruitment of R1 and R6 photoreceptors appeared to be delayed (data not shown), with Bar positive cells not appearing until at least the 12^th ^ommatidial row posterior to the furrow, when normally Bar positive cells can be observed approximately 5 rows posterior to the furrow (for example see fig. 6C).

Analysis of R7-cell development in *lz*^Gal4^, *UAS-gfp*; *UAS-ttk*^RNAi^; *sina*^-^*msi*^- ^eye discs using Runt as a marker revealed the development of ectopic, *lz*-expressing R7 cells in most developing ommatidia (fig. 8H-H'; 92%, N = 402), a phenotype already attributable to the loss of Ttk69 function in eye development (see fig. 3). Taken together, these results confirm that the loss of R1, R6 and R7 cells in *sina msi *double mutants can be largely attributed to the failure of Ttk degradation, and show conclusively that Sina and Msi function redundantly to regulate Ttk expression in R1 and R6 cells. Furthermore, the rescue of *lz *expression in all *lz*-dependent cells adds extra weight to the hypothesis that *lz *can be negatively regulated by Ttk69 in the developing eye, either directly or indirectly.

## Discussion

The *Drosophila *Ttk69 transcriptional repressor has previously been shown to play a critical role in a number of developmental processes, including specification of glia in the embryonic CNS [62], photoreceptor differentiation in the eye [19,24,26], and dorsal follicle cell migration and chorion production in the ovary [63]. These developmental roles for Ttk69 have been shown to be dependent upon the repressive activity of Ttk69. Here, we have established an interaction between *ttk69 *and *lz *mutant alleles and have shown that Ttk69 can repress *lz *expression in the third instar developing eye disc. Furthermore, we have shown that the development of ectopic R7 cells in *ttk *loss of function mutants is dependent upon the function of Lz. Our results have led us to conclude that Ttk69 may repress *lz *in a subset of precursor cells competent to develop as R7 cells. One important finding from our studies was that upon overexpression of Ttk69, Lz protein expression was observed in a wave of cells posterior to the furrow prior to its reduction in basally located undifferentiated cells in the posterior portion of the disc (fig. 6J). This result could reflect a delay in the onset of producing Ttk69 at levels required to repress Lz expression, or alternatively, it could highlight the ability of Ttk69 to repress *lz *in a subset of cells in early eye development.

Loss of Ttk69 expression in third instar eye epithelia resulted in development of ectopic *lz*-expressing presumptive R7 cells, revealing that only precursor cells competent to develop as R7's were affected by the loss of Ttk69 function. Overexpression of Ttk69 caused severe reduction of *lz *expression in all *lz*-dependent differentiating cell types as measured by the *lz*^*Gal*4 ^driven expression of GFP, while re-introduction of the *GMR-lz *transgene resulted in the significant rescue of 69% of *lz*-dependent R7 cells. Interestingly, only 32% of R1 or R6 cells could be rescued, suggesting that genes, other than *lz*, are necessary for development of these cells and were affected by Ttk69 overexpression in the developing eye. Additionally, Lz protein expression was down-regulated upon overexpression of Ttk69 in both undifferentiated and differentiated cell types. Taken together, we hypothesise that Ttk69 repression of *lz *in eye development functions in a set of cells to control the specificity and level of *lz *gene expression in order to correctly specify the R7 cell.

Further support for our hypothesis comes from analysis of *sina *and *msi *mutants. Sina has been proposed to negatively regulate both Ttk isoforms in *Drosophila *eye development [19-21]. Additionally, Msi has been shown to translationally repress *ttk69 *mRNA in developing sensory organ precursor cells of the neuroectoderm, with mutations in *msi *resulting in bristle duplication forming from the one socket [28]. With respect to eye development, Hirota and colleagues [27] proposed that Sina and Msi could function redundantly in presumptive R1 and R6 cells to negatively regulate Ttk69 expression. Our findings have supported this model, with knockdown of Ttk expression in a *sina msi *double mutant background resulting in the rescue of the majority of R1 and R6 cells. The recovery of only 73% of presumptive R1 and R6 cells in *lz*^Gal4^, *UAS-gfp*; *UAS-ttk*^RNAi^; *sina*^-^*msi*^- ^eye discs could be due to a failure of full knockdown of the Ttk gene product by expressing the RNAi transgene, or due to a secondary but as yet uncharacterised role for Sina in R1 and R6 photoreceptor development. Importantly, ectopic *lz*-expressing R7 cells were observed in almost all of *lz*^Gal4^, *UAS-gfp*; *UAS-ttk*^RNAi^; *sina*^-^*msi*^- ^mutant ommatidia, demonstrating the significance of the inhibitory effect of Ttk in R7 cell regulation.

**Figure 9:**
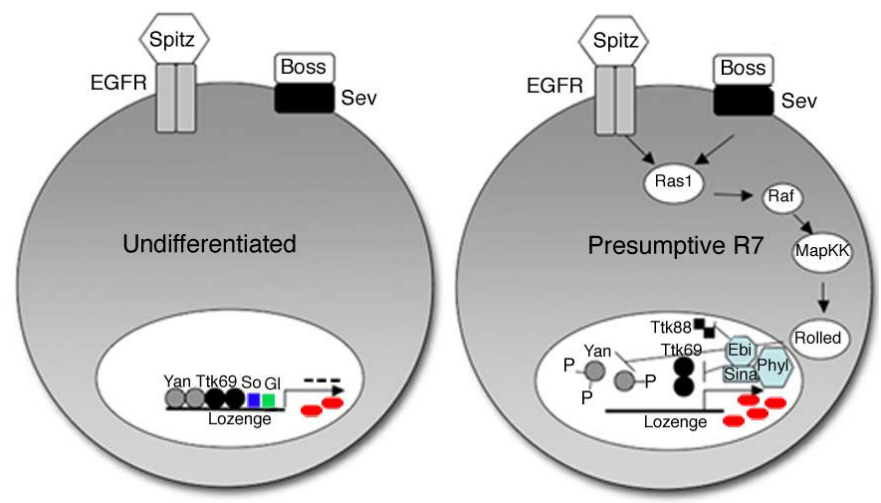
**Model for Ttk69 regulation of *lz *in *Drosophila *eye development**. Model for the regulation of *lz *based upon data from this study, Behan and colleagues [39,41] and Yan and colleagues [33]. In undifferentiated cells, So and Gl initiate the expression of *lz *(red oval represents Lz protein product). Yan and Ttk69, both transcriptional repressors, are required to down-regulate expression of *lz *in undifferentiated cells, keeping Lz levels at an appropriate level. Upon activation of the Ras1/MapK signal transduction cascade, the repressive effects of Yan and Ttk69 on *lz *expression are relieved, thus enabling cell fate specification events in the R7 cell to proceed.

Ttk69 is a downstream target of Ras1/MAPK signalling, and Ttk isoforms are targeted for degradation upon induction of this signalling cascade [19,21,64]. Our conclusion that Ttk69 negatively regulates *lz *gene expression implies that induction of the Ras1/MAPK signalling cascade leads to the de-repression of *lz *due to degradation of the Ttk protein. Although initial reports suggested that *lz *was not a downstream target of the Ras1 signalling pathway [34], a more recent study from our laboratories have shown a genetic interaction between *ras1 *and *lz *alleles [39]. This latter study showed that the mild rough eye phenotype caused by expression of a dominant negative Ras1 allele from the *sev *promoter (*sev-*Ras^N17^) could be enhanced in flies where half the dose of *lz *was removed. Additionally, expression of a constitutively active Ras1 allele under the control of *sev *(*sev-ras1*^*val*12^) could partially rescue adult eye defects observed in *lz *temperature sensitive mutants [39]. The study further demonstrated that Yan, an inhibitor of RTK signalling in the *Drosophila *eye [24,65,66], can negatively regulate *lz *in a subset of cells in early eye development, with the conclusion being that Yan represses *lz *expression in undifferentiated cells, and as cells differentiate, *lz *expression increases due to the removal of Yan's influence on *lz *repression [39]. Interestingly, this repression also appears to occur in precursor cells competent to develop as R7 cells, with loss of *yan *function causing the conversion of mystery cells to ectopic, *lz*-expressing R7 cells [39]. The co-operative action of Yan and Ttk69 in repressing R7 photoreceptor cell fate has previously been reported [67], as the loss of *yan *function in the developing eye results in the development of ectopic R7 cells, and this phenotype can be dominantly enhanced by the reduction of *ttk69 *function [29,67]. Therefore, it is likely that Yan and Ttk69 play redundant or synergistic roles in *lz *gene repression in undifferentiated cells of the eye (fig. 9).

Ttk69 normally exerts its repressive effects by direct binding to core sites in promoter regions of target genes. For example, Ttk69 can compete with PointedP2 for binding the *string *promoter, thus playing a part in regulating the second mitotic wave in eye morphogenesis [46]. Functional binding sites have also been identified in other Ttk69 target genes, including *tailless *[25], *fushi tarazu *[68], and *even skipped *[69]. In our study, we have identified putative core Ttk69 binding sites in the *lz *eye enhancer region, and these binding sites are conserved across three *Drosophila *species. The *lz *eye enhancer has been shown to be essential for mediating the regulation of *lz *expression in the eye, in particular, providing a molecular target for activation of *lz *expression in undifferentiated cells posterior to the furrow by two proteins, So and Gl [33,38]. Interestingly, So and Gl binding sites are also highly conserved across different *Drosophila *species, and are present in the same enhancer region as the Ttk69 sites. In addition, an ETS (Yan/PntP2) binding site is also highly conserved in this region. Our previous studies have indicated that Yan represses *lz *in eye development [39], and another study has shown that PntP2 and Ttk69 can compete for direct binding to the *string *promoter [46]. While we have not in this study demonstrated the direct binding of Ttk69 to the *lz *eye enhancer region, it remains plausible that the repression of *lz *by Ttk69 is direct.

In addition to its expression in undifferentiated cells, Ttk69 is also expressed in cone cells in the third instar eye epithelia, and in photoreceptor cells later in pupal development [26]. This pattern of expression correlates with its function, since Ttk69 is known to repress the neuronal fate but promote non-neuronal fate induction in third instar eye development [19,21,23,24], while Ttk69 plays a positive role in photoreceptor development in pupal eye development [26]. However, the developmental role of Ttk69 in this context is likely to depend on the levels of Ttk69 expression. For example, while Ttk69 is thought to induce non-neuronal cell fates, we and others have shown that high levels of Ttk69 expression driven from either the *sev*-Gal4 driver, or the *GMR*-Gal4 driver, can inhibit both photoreceptor development and cone cell development (fig. 7; [19,29]). Importantly, Ttk69 is not normally degraded in presumptive cone cells, yet *lz *expression persists in these cells, highlighting that appropriate levels of transcription factors are critical in defining the combinations of genes expressed in particular cell types. It is possible that a low titre of Ttk69 in cone cells can partially repress expression of *lz*, thus differentiating them from presumptive R7 cells. Indeed, R7 and cone cells arise from a developmentally equipotent group of cells known as the R7 equivalence group, and therefore subtle changes within this group of cells are necessary to mediate the correct fate outcomes [65,70,71]. Alternatively, Ttk69 binding partners may be absent in cone cells, therefore impeding the ability of Ttk69 to exert it repressive effect. Whatever the case, the mechanism by which Ttk69 can promote the differentiation of some cell types, yet inhibit others is currently unclear.

## Conclusion

Cell fate induction in the developing eye is dependant upon the expression of unique sets of transcription factors in a spatial and temporal manner [72]. Lz plays an important role as a pre-patterning factor in early eye development, since its expression in undifferentiated cells is necessary for the recruitment and subsequent differentiation of neuronal R1, R6 and R7 cells, and non-neuronal cone and pigment cells. Furthermore, Lz acts in combinatorial manner with a number of factors, in a cell specific manner, to transcriptionally activate, or repress, transcription factors expressed after the second mitotic wave [32,34,38,40,41,43]. It is therefore critical that *lz *itself is tightly regulated in differentiating cells. This regulatory constriction must extend to the level of *lz *expression, since expression levels of a gene can greatly influence the specification of individual cell types. For example, functional analysis of the cell fate specification gene *seven-up *in the developing eye epithelia revealed that if ectopic expression levels of Svp are low in R7 and cone cell precursors, cone cells will be converted to R7 cells, whereas if levels are too high, R7 and cone cells will adopt an outer photoreceptor fate [73-75]. It is therefore not surprising to consider that Ttk69 may, in part, regulate the levels of *lz *expression in a specific pool of equipotent cells to allow for the correct fate specification of cells specified after the second mitotic wave.

Lz is a member of the Runx family of transcription factors, and these highly conserved proteins play key roles in the regulation of a number of developmental processes such as epithelial development, hematopoesis and neurogenesis in mammals [76] and in eye development and hematopoesis in flies [77]. Similarities between mammalian Runx and *Drosophila *Lz proteins extend to the level of gene regulation and protein-protein interaction, with both Runx1 and Lz being alternatively spliced to remove an ETS interaction domain, and both proteins having the ability to physically interact with the ETS-1 transcription factor [41,78]. Tramtrack69 is a member of the BTB/POZF family of transcription factors, a diverse group of proteins functioning in many biological processes, including cell cycle progression, B cell fate determination and hematopoietic stem cell fate determination [79]. The human genome encodes approximately 60 POZF proteins [79], with at least one of these proteins having been demonstrated to be involved in antagonising Runx activity in T cell fate specification [80]. Therefore, the elucidation of mechanisms and factors involved in Lz regulation in *Drosophila *will greatly enhance our understanding of Runx regulation in key mammalian developmental processes.

## Methods

### *Drosophila *strains

Most *lz *stocks were obtained from Bloomington Stock Center, Indiana, Mel Green (University of California at Davis) and Reinhard Stocker (University of Fribourg, Switzerland). *GMR-lz c3.5 *was obtained from Utpal Banerjee (U.C.L.A). The origin of the *lz*^*Gal*4 ^enhancer trap line has been previously described [39,40]. The *ttk *alleles, *FRT82B ttk*^1*e*11 ^and *frt82B ttk*^*rm*730 ^[26], were obtained from Z.-C. Lai (Penn. State University). The *ttk*^*RNAi *^line was obtained from the Vienna *Drosophila *RNAi centre. *sina*^2 ^and *sina*^3 ^alleles were obtained from the G. Rubin laboratory (Howard Hughes Medical Institute), and the *sina msi *combination alleles [27] were obtained from M. Okabe (National Institute of Genetics, Japan). The *klingon-lacZ *line B38 was obtained from Yasushi Hiromi (National Institute of Genetics, Japan). All *ey*Flp strains used to create mosaic animals were obtained from B. Dickson (Institute of Molecular Pathology, Vienna), or from the Bloomington stock centre. All other strains were obtained from Bloomington stock centre with the exception of the UAS-*ttk69 *strain, which was obtained from H. Richardson (Peter MacCallum Cancer Institute, Aus). All strains were reared on standard corn meal molasses or standard laboratory medium at 25°C.

### Mosaic analysis and genetics

*ttk*^1*e*11 ^and *ttk*^*rm*730 ^clones were induced by the *ey*FLP technique [48]. The eye clones were induced in flies of genotype *y w ey*FLP1; FRT82B 3R3.7 P [*w*^+^, *arm*-*lacZ*]/FRT82 *ttk*^1*e*11 ^(or FRT82 *ttk*^*rm*730^). Eye clones in *lz *backgrounds were also induced in the following genotypes: *lz*^*Gal*4^, UAS-GFP/*y w ey*FLP1; FRT82B 3R3.7 P [*w*^+^, *arm*-*lacZ*]/FRT82B *ttk*^- ^and *lz*^*mr*1^/Y (or *lz*^*mr*2^/Y); *ey*FLP1; FRT82B 3R3.7 P [*w*^+^, *arm*- *lacZ*]/FRT82B *ttk*^-^. The *white*^+ ^marker, located distal to the FRT on the non-*ttk *mutant chromosome, was used to identify *ttk*^- ^clones in adults, with homozygous *ttk*^- ^tissue being *w*^-^. Mosaic clones were further selected for by the addition of a cell lethal mutation (3R3.7) into the system. The twin-spot cells that are homozygous for the 3R3.7 mutation, but not the *ttk*^- ^mutation, die. This leaves only *ttk*^- ^homozygous cells and non-recombinant heterozygous cells. As a result, *ttk*^- ^clones occupied the majority of the eye [48]. Clones in the developing larval eye disc were initially characterised by a lack of β-galactosidase staining, since the *arm-lacZ *construct was located distal to the FRT on the non-*ttk *mutant chromosome. For some experiments, we also created *ttk *clones in flies of genotype *ey*Gal4 UAS-Flp; FRT82B UbiGFP/FRT82B *ttk*^1*e*11 ^(or FRT82B *ttk*^*rm*730^). These flies lacked a cell lethal mutation enabling the direct comparison between wildtype GFP positive clonal patches, and negatively marked mutant clonal patches.

Genetic and immunohistochemical analysis was also carried out in the following genotypes: *lz*^*Gal*4^, UAS-GFP/Y;UAS-*ttk69*/+, *lz*^*Gal*4^, UAS-GFP/Y;UAS-*ttk69*/GMR-*lz c3.5*, *lz*^*Gal*4^, UAS-GFP/Y;UAS-*ttk69*/GMR-*lz c3.5B38, lz*^*Gal*4^, UAS-GFP/Y;*sina*^2^/*sina*^3^, *lz*^*Gal*4^, UAS-GFP/Y; *sina*^2^*msi*^1^/*sina*^3^*msi*^1 ^and *lz*^*Gal*4^, UAS-GFP/Y; UAS-*ttk*^*RNAi*^; *sina*^2^*msi*^1^*ttk*^*osn*^/*sina*^3^*msi*^1^. To make the *GMR-lz c3.5 B38 *strain, *GMR-lz c3.5 *and B38 flies were recombined using standard genetic techniques.

### Antibody staining and microscopy

For immunohistochemical analysis of third instar larval eye imaginal discs, flies were reared at 25°C and collected for dissection at crawling third instar stage of development. In most cases, males were selected for analysis on the basis of the presence of gonads, which appear as transparent bodies on each side of the fifth abdominal segment. Eye-antennal imaginal discs were removed, fixed in 4% Paraformaldehyde for 30 minutes, washed in 1 × phosphate buffer solution (PBS) 3 times for 10 minutes each, and permeabilised in 0.2% PBT (PBS with 0.2% Triton-X 100). The appropriate primary antibodies were then diluted in PBTN (PBT with 5% natural goat serum) and the tissue was incubated for 2 hours at room temperature, or overnight at 4°C. The tissue was washed 3 times for 10 minutes and then incubated in secondary antibody (diluted in PBTN) for 1 hour 30 minutes at room temperature. Before rinsing in 1 × PBS and mounted in Vectashield^® ^mounting media on frosted slides. Primary antibodies used in this study included Rat anti-Elav (1:200, developed by G. Rubin, obtained from the Developmental Studies Hybridoma Bank (DSHB), Iowa), Mouse anti-Prospero (1:200, DSHB), Mouse anti-Cut (1:200, DSHB), Rabbit anti-Bar (1:50, obtained from K. Saigo, Tokyo University), Guinea-pig anti-RUNT (1:200, obtained from R. Saint, Australian National University, Aus, Asian Distribution Centre for segmentation antibodies), Rat anti-E-cadherin (1:20, DSHB), Rabbit anti-β galactosidase (1:500, Chemicon), and Mouse anti-Lozenge (1:10, DSHB). For the Lz stain, peripodial membranes were removed prior to staining, and discs were permeabilised in 0.3% PBT with 0.3% Saponin and 0.3% Sodium Deoxycholate. All secondary antibodies were Alexa-fluor conjugates obtained from Molecular Probes Inc., and were all used at a dilution of 1:500. The Optiscan F900e confocal system and Zeiss Meta confocal were both used to image and examine samples. Images were processed using Adobe Photoshop 6.0 software.

Scanning electron microscopy of 0-3 day old flies was achieved as described in [81]. Images were digitally acquired using Spectrum software. Alternatively, unfixed 0-3 day old flies were imaged using a Hitachi S-2460N.

### Bionformatics

The multiple sequence alignment was performed in MacVector™7.1 using slow clustalW, with an open gap penalty of 3.0 and an extended gap penalty of 0.5. These parameters were chosen to account for the high level of insertion/deletion (indel) events.

## Authors' contributions

NAS carried out and designed the studies, analysed the data and drafted the manuscript. GRH participated in drafting the manuscript and contributed to the analysis of some data. JAP participated in the design and analysis of this project and also participated in the drafting of the manuscript. PB participated in the design and analysis of this project. All authors have read and approved the final manuscript.
